# Migration background and specialized mental health care utilization in young adults: a register-based study in the Netherlands

**DOI:** 10.1007/s00127-026-03052-0

**Published:** 2026-02-11

**Authors:** Cansu Alozkan Sever, Caterina Ceccarelli, Pim Cuijpers, Ellenor Mittendorfer-Rutz, Jelger P. van Dam, Aemal Akhtar, Oscar Oelrich, Els van der Ven, Anke B. Witteveen, Marit Sijbrandij

**Affiliations:** 1 Department of Clinical, Neuro- and Developmental Psychology, WHO Collaborating Center for Research and Dissemination of Psychological Interventions, Vrije Universiteit Amsterdam, Van der Boechorststraat 7, 1081 BT, Amsterdam, The Netherlands; 2 Amsterdam Public Health Research Institute, Amsterdam, The Netherlands; 3 Babeș-Bolyai University, International Institute for Psychotherapy, Cluj-Napoca, Romania; 4 Division of Insurance Medicine, Department of Clinical Neuroscience, Karolinska Institutet, Stockholm, Sweden

**Keywords:** Migration, Young adults, Epidemiology, Anxiety disorder, Psychotic disorder, Bipolar disorder

## Abstract

**Purpose:**

This study aimed to examine the differences in specialized mental health care utilization among young conflict-affected migrants in the Netherlands, compared to their Dutch-born and non-conflict-affected peers, and to assess the role of benefit receipt and duration of stay.

**Methods:**

This register-based study included 1,219,051 individuals aged 18–25 residing in the Netherlands on 1 January 2015. Participants were followed until 31 December 2018 for specialized mental health care contact. Adjusted hazard ratios (aHR) with 95% confidence intervals (CI) were estimated using Cox regression. Exploratory analyses examined how benefit receipt and duration of stay relate to specialized mental health service use.

**Results:**

Compared to Dutch-born individuals, both conflict-affected (aHR 0.91, 95%CI 0.88–0.95) and non-conflict-affected migrants (aHR 0.63, 95%CI 0.61–0.65) had lower overall specialized mental health care utilization. Conflict-affected migrants showed higher utilization for psychotic (aHR 3.99, 95%CI 2.39–4.69) and anxiety disorders (aHR 1.27, 95%CI 1.17–1.38), whereas non-conflict-affected migrants had higher utilization for psychotic disorders (aHR 2.03, 95%CI 1.72–2.34) compared to Dutch-borns. Patterns of utilization also varied by benefit receipt and duration of stay, with differences across diagnostic categories.

**Conclusion:**

Young migrants had lower overall use of specialized mental health care than Dutch-born peers, but higher use for psychotic, and anxiety disorders, varying by migration background. As unmet need cannot be assessed from our data, it remains unclear whether lower use reflects reduced need or access barriers. However, findings underscore persistent disparities and the need for policies ensuring equitable access.

**Supplementary Information:**

The online version contains supplementary material available at https://doi.org/10.1007/s00127-026-03052-0.

Young adulthood is a critical period for mental health, accounting for 45% of the burden of disease among people aged 10–24 globally [1], and 62.5% of mental health disorders emerge before the age of 25 [2]. However, most mental health disorders emerging by age 18 remain undetected and untreated [3]. Young people with a migration background face an even greater risk of mental health disorders [4], and exposure to wars and conflicts may further exacerbate these vulnerabilities [5].

Traumatic experiences, migration-related difficulties, and other social risk factors have been found to contribute to negative mental health outcomes among migrants [4]. It is consistently reported that young refugees face elevated rates of mental health disorders, particularly posttraumatic stress disorder (PTSD), depression, and anxiety disorders [6–8]. Similarly, young non-refugee migrants face a heightened risk of psychotic disorders, psychosomatic conditions, and substance use disorders compared to non-migrant populations [4]. Despite these elevated risks, evidence on incidence rates and mental health care utilization among young refugees and migrants remains limited. Even though several studies reported on the possible barriers to mental health care use for refugees in general [9, 10] and among young refugees [11], it remains unclear to what extent these barriers translate into lower rates of service utilization. Studying the incidence of specialized mental health care use can help reveal whether elevated mental health needs among young migrants are met with appropriate care.

Population-based studies from Scandinavian countries offer valuable insights into mental health care use among young migrants. A Swedish registry study found that young refugees and non-refugee migrants were less likely to use psychiatric services than their Swedish-born peers, except for psychotic and stress-related disorders [12]. Similarly, Danish cohort studies reported lower overall psychiatric service use among refugee children and non-refugee migrants compared to Danish-born youth, with higher rates observed specifically for psychotic and nervous disorders in early adulthood [11, 13]. A binational study using registries from Sweden and Denmark further showed elevated incidence rates of non-affective psychotic disorders among non-majority groups, although differences between refugees, non-refugee migrants, and descendants of migrants were relatively small [14]. These findings suggest that patterns of specialized mental health care use among young migrants may vary by disorder, highlighting the need to better understand how and when different migrant groups utilize care.

Despite these insights from Scandinavian countries, comparable population-based evidence from the Netherlands remains limited. Although nearly 15% of the Dutch population is foreign-born [15], large-scale national studies on the incidence of specialized mental health care among migrants are scarce. Existing Dutch registry-based research has primarily focused on specific regions or diagnoses, with evidence suggesting higher incidence rates for schizophrenia among migrants from particular countries (e.g. Surinam, Morrocco) compared to Dutch-borns [16–18], as well as higher treatment risks for depressive and non-affective psychotic disorder among non-Western migrants compared to Western European migrants, except for bipolar disorder [19].

As noted above, existing evidence on mental health care utilization among migrants is mixed, with considerable variation across different migrant subgroups. Previous studies have sought to explain this heterogeneity by examining several factors. One such factor is labor market marginalization (LMM), and research showed that refugees face a heightened risk of LMM compared to non-migrant populations [20]. In the Dutch context, receiving unemployment, disability, or social assistance benefits is closely linked to LMM, as these forms of support typically indicate limited or no participation in paid employment. Studies showed that in the Netherlands, social assistance recipients, in particular, tend to have the weakest attachment to the labor market [21]. Mental health challenges during adolescence are also associated with reduced labor market participation in young adulthood, as demonstrated by a Dutch prospective cohort study that followed participants for 15 years [22]. Other evidence suggests that this relationship might be bidirectional: exclusion from the labor market can also contribute to the onset or worsening of mental health problems, potentially reinforcing a cycle of marginalization [20, 23].

Patterns of mental health care use among refugees and migrants also varied by duration of stay in the host country. Evidence suggests that psychiatric care utilization increases with longer time spent in the host country. Manhica and colleagues found that psychiatric care use among refugee youth increased with length of stay in Sweden [24]. Similarly, another study reported that newly resettled refugee youth (≤ 5 years) were less likely to use psychiatric health care than those who had resided in the country for over 10 years [12]. However, disorder-specific trends may differ; use of care for stress-related disorders such as PTSD tends to decrease over time [25]. In the Netherlands, a large cohort study using antipsychotic medication prescription data and insurance claims found that the risk of psychosis-related treatment was initially low after arrival but increased in subsequent years, especially among migrants from non-Western countries [26]. These patterns may reflect delayed access, changing needs, or adaptation processes, but the underlying mechanisms remain uncertain and call for further investigation.

## Aim of the study

This study examined the differences in the incidence of specialized mental health care use among young migrants from conflict-affected and non-conflict-affected countries compared to their Dutch-born peers, utilizing population-based registry data from Statistics Netherlands. It also explored the associations between migration characteristics and benefit receipt, with specialized mental health care utilization. To our knowledge, this is the first national study in the Netherlands that investigates these factors among young adults using registry data.

## Methods

### Data source

This national registry-based study used de-identified data from Statistics Netherlands (in Dutch: Central Bureau of Statistics; CBS), accessed with permission. Unique identifiers linked six datasets: (1) municipal registration with birth details and parents’ country of birth [27]; (2) migration background with immigration/emigration dates [28]; (3) mortality data [29]; (4) education records to identify the highest attained education [30]; (5) primary income source to classify labor market position [31]; and (6) specialized mental health care utilization from health insurance claims for mental health care trajectories [32]. These trajectories are opened when a patient requests care from an institution/practice and include combined information of received diagnoses and treatments provided. Mental health care data covered 2015 to 2018, as completeness declined after 2018 due to changes in the Health Insurance Act [33].

### Study population

The study population included individuals aged 18–25 years living in the Netherlands on January 1, 2015 (*n* = 1,892,365). We excluded those not residing in the Netherlands at baseline (*n* = 143,049), those who had died before baseline (*n* = 8,509), migrants residing in the Netherlands for less than three years (*n* = 64,543), individuals younger than 18 at baseline (*n* = 62,669), and second-generation migrants (born in the Netherlands to one or both foreign-born parents; *n* = 298,567). We also excluded individuals with incomplete migration data and those with prior contact with specialized mental health care (*n* = 95,977). The final cohort included 1,219,051 individuals: 33,224 born in conflict-affected countries, 85,063 in non-conflict-affected countries, and 1,100,764 in the Netherlands.

### Outcome

The primary outcome of this study is the initiation of specialized mental health care. We identified the first mental health contact of each individual and the associated main diagnosis. Diagnostic information was based on the DBC Information System (DIS) used in Dutch mental health care, which follows the Diagnostic and Statistical Manual of Mental Disorders IV (DSM-IV) [34]. Follow-up was conducted from January 1, 2015, to December 31, 2018. In addition to receiving any psychiatric diagnoses, the following diagnoses were studied separately: depressive disorders, anxiety disorders (including PTSD), bipolar disorders, psychotic disorders, alcohol-related disorders, substance-related disorders, adjustment disorders, and somatoform disorders (DIS codes and the list of diagnoses can be found in Supplementary Table 6).

### Exposure variables

#### Migration status

Migration status was categorized into three groups. *Dutch-born* individuals were those born in the Netherlands to parents also born in the Netherlands. *Conflict-affected migrants* were born in countries experiencing war or armed conflict. Classification was based on migration records indicating asylum as a motive, supplemented by the Uppsala Conflict Data Program of Uppsala University^1^, (UCDP), a widely used source identifying the countries’ conflict status in health and migration research [35]. We defined conflict status using the individual’s country of birth and the conflict status of that country at the time of their birth (see Supplementary Table 5). *Non-conflict-affected migrants* were born in countries not affected by conflict and migrated to the Netherlands mainly for work, study, or family reunification.

#### Migration characteristics

*Duration of stay in the Netherlands* were calculated using the baseline date and the arrival date in the Netherlands. This variable was categorized into three categories: *3–5 years*, *6–10 years*, and *over 10 years*.

#### Socioeconomic variables

*Benefit receipt* was based on each individual’s main income source at baseline, determined by the highest amount if multiple sources existed. We created a binary variable: those whose primary income was a benefit were coded as 1, others as 0 (see Supplementary Table 7). This variable served to assess labor market status, specifically whether individuals relied on benefits.

### Covariates

We adjusted the models for age and sex. Additionally, we adjusted for country of birth in one of the sensitivity analyses. Country of birth has been grouped as the *Netherlands*,* Africa*,* Asia (except the Middle East)*,* Europe*,* Former Soviet Union*,* Former Yugoslavia*,* Middle East*, and *Other Countries*.

### Statistical analyses

All analyses were conducted in the CBS microdata remote working environment using R statistical software (version 4.0.1) and the R packages *dplyr* [36], *survival* [37], and *car* [38]. The primary analyses examined the association between the utilization of specialized mental health care and migration background. Crude and multivariate analyses were performed using Cox regression models to estimate the time to the first recorded specialized mental health care contact during the follow-up period. The results are reported as crude (HR) and adjusted hazard ratios (aHR) with 95% confidence intervals (CI). Person-years at risk were calculated by adding up the amount of time each individual was followed during the study period. The entry date was January 1, 2015, and follow-up ended on December 31, 2018, or earlier in the event of mental health care utilization, death, or emigration. Separate analyses were performed for each diagnostic group. Model 2 was adjusted for age and sex. We evaluated the proportional hazards assumption by estimating Schoenfeld residuals and examining statistical tests and residual plots. To address multiple comparisons, we applied a False Discovery Rate (FDR) correction for the primary Cox model, which included all diagnostic categories. No multiplicity correction was performed for the explanatory analyses, consistent with their exploratory purpose. Results are based on calculations by the authors using non-public microdata from Statistics Netherlands. According to the microdata usage rules of Statistics Netherlands, a statistical finding can be published only if it is based on more than 10 individual records.

### Sensitivity analyses

We conducted sensitivity analyses to test the robustness of our findings. First, we excluded migrants from high-income countries, focusing on individuals from low- and middle-income countries (LMICs) based on World Bank classification [39], and removed Dutch-born individuals to compare the two migrant groups. Lastly, we excluded non-emergency psychiatric diagnoses and repeated both crude and adjusted analyses.

## Results

### Characteristics of the study population

Table 1 summarizes the study population by migration background. The sex distribution of the cohort was nearly equal, with 48.44% women and 51.56% men. The mean age was 21.34 years in the whole population. Secondary education was the most common, while university-level education was higher among Dutch-born individuals (16.78%) compared to conflict-affected (11.32%) and non-conflict-affected migrants (7.34%). Missing education data were most frequent among non-conflict-affected migrants (42.80%). Most conflict-affected migrants came from the Middle East (45.67%) and Africa (30.36%), while non-conflict-affected migrants were primarily from Europe (53.99%). Conflict-affected migrants arrived younger (12.60, ± 6.74) than non-conflict-affected migrants (17.74, ± 6.18) and had the lowest employment rate (14.34%) and the highest proportion of individuals whose primary source of income was benefits (at least 15.1%, excluding pension benefits). Apart from the highest education attained, missing data for baseline sociodemographic variables were negligible, with rates below 0.2% (See Table 1 for detailed characteristics).

**Table 1:** Cohort characteristics, by migration background, in individuals aged 18–25 years old residing in the Netherlands in 2015

	Dutch-born individuals	Conflict-Affected Migrants	Non-Conflict Affected Migrants
All (*n*)	1,100,764	33,224	85,063
**Baseline Sociodemographics**			
*Sex (n*,* %)*			
Women	536,798 (48.77%)	15,641 (47.08%)	38,092 (55.22%)
Men	563,966 (51.23%)	17,583 (52.92%)	46,971 (44.78%)
Mean age (years, s.d.)	21.07 (2.04)	21.32 (2.03)	21.64 (1.93%)
*Highest attained Education (n*,* %)*			
Primary school	23,798 (2.16%)	4,628 (13.93%)	4,065 (4.78%)
Secondary school	863,157 (78.41%)	21,392 (64.39%)	34,957 (41.10%)
University or higher	184,718 (16.78%)	2,440 (7.34%)	9,633 (11.32%)
Missing	29,091 (2.64%)	4,764 (14.34%)	36,408 (42.80%)
*Country of Birth (n*,* %)*			
Netherlands	1,100,764 (100%)	–	–
Africa	–	10,087 (30.36%)	4,612 (5.42%)
Asia (except Middle East)	–	997 (3.00%)	18,704 (21.99%)
Europe	–	–	45,927 (53.99%)
Former Soviet Union	–	4,498 (13.54%)	3,367 (3.96%)
Former Yugoslavia	x*	x	x
Middle East	–	15,173 (45.67%)	667 (0.78%)
Others	x	x	x
Mean age of arrival (years, s.d.)	–	12.60 (6.74%)	17.74 (6.18%)
*Duration of stay in the NL (n*,* %)*			
3–5 years	–	12,360 (37.20%)	62,229 (73.16%)
6–10 years	–	4,508 (13.57%)	7,437 (8.74%)
More than 10 years	–	16,356 (49.23%)	15,397 (18.10%)
*Main source of income (n*,* %)*			
Employment	404,617 (36.76%)	5,916 (17.81%)	20,253 (23.81%)
Unemployment benefits	9,869 (0.90%)	176 (0.53%)	879 (1.03%)
Social benefits	44,650 (4.06%)	4,776 (14.38%)	1,856 (2.18%)
Illness/disability benefits	3,731 (0.34%)	64 (0.19%)	339 (0.40%)
Pension benefits	x	x	x
Student or not yet in school	570,346 (51.81%)	16,393 (49.34%)	32,763 (38.52%)
Other without income	46,479 (4.22%)	5,890 (17.73%)	28,942 (34.02%)
Missing	x	x	x

*Some results are not reported due to low occurrences, in line with CBS Microdata usage rules

### Differences in specialized mental health care utilization by migration background

Table 2 presents the results of primary Cox regression analyses examining the association between migration background and specialized mental health care utilization. During the follow-up period, 8.11% (*n* = 89,298) of Dutch-born individuals, 6.80% (*n* = 2,260) of conflict-affected migrants, and 3.74% (*n* = 3,183) of non-conflict-affected migrants utilized specialized mental health care. Non-conflict-affected migrants had the lowest rate for overall utilization (141.10 per 10.000 person-years). The risk of using specialized mental health care services among both migrant groups was significantly lower than the reference population (aHR 0.91, 95% CI 0.88–0.95 for conflict-affected migrants; aHR 0.63, 95% CI 0.61–0.65 for non-conflict-affected migrants).

For specific diagnostic categories, conflict-affected migrants had a higher risk of utilization for anxiety disorders (aHR 1.27, 95% CI 1.17–1.38) and psychotic disorders (aHR 3.99, 95% CI 3.39–4.69) compared to the Dutch-born population. In contrast, they had a lower risk of utilization for alcohol use disorders (aHR 0.73, 95% CI 0.49–1.09), substance-related disorders (aHR 0.68, 95% CI 0.55–0.84), and somatoform disorders (aHR 0.63, 95% CI 0.47–0.83).

Compared to Dutch-born young adults, non-conflict-affected migrants had a lower risk of utilization for most of the diagnoses, including depressive disorders (aHR 0.72, 95% CI 0.66–0.78), anxiety disorders (aHR 0.68, 95% CI 0.63–0.73), and somatoform disorders (aHR 0.23, 95% CI 0.17–0.32). Like conflict-affected migrants, they had a higher risk of utilization for psychotic disorders (aHR 2.03, 95% CI 1.72–2.34). For bipolar disorders, estimates suggested a modest elevation in utilization, but with substantial uncertainty (aHR 1.20, 95% CI 0.95–1.53) compared to the reference group.

For the primary Cox regression models, multiplicity was addressed using False Discovery Rate (FDR) correction across diagnostic categories; all conclusions remained unchanged after adjustment (Supplementary Table 1). The proportional hazards assumption was assessed using Schoenfeld residual plots and statistical tests. Visual inspection showed no evidence of violation across any of the models. Although two outcomes yielded statistically significant test results, the corresponding plots displayed only minimal, non-systematic trends, which we consider unlikely to indicate meaningful departures from proportionality given the very large sample size.

**Table 2:** Specialized mental health care utilization, by migration background, individuals aged 18–25 residing in the Netherlands in 2015. Hazard ratios (HRs) with 95% confidence intervals (CIs)

	*N* (rate per 10,000 person-years)	Model 1^a^	Model 2^b^
*Any diagnosis*			
Dutch-born	89,298 (212.49)	1 (REF)	1 (REF)
Conflict affected	2,260 (194.51)	0.91 (0.87–0.95)	0.91 (0.88–0.95)
Non-conflict affected	3,183 (141.10)	0.65 (0.62–0.67)	0.63 (0.61–0.65)
*Depressive Disorders*			
Dutch-born	14,902 (35.46)	1 (REF)	1 (REF)
Conflict affected	382 (32.88)	0.92 (0.84–1.02)	0.93 (0.84–1.03)
Non-conflict affected	608 (26.95)	0.75 (0.69–0.82)	0.72 (0.66–0.78)
*Anxiety Disorders*			
Dutch-born	17,642 (41.98)	1 (REF)	1 (REF)
Conflict affected	616 (53.02)	1.26 (1.16–1.36)	1.27 (1.17–1.38)
Non-conflict affected	697 (30.90)	0.72 (0.67–0.78)	0.68 (0.63–0.73)
*Bipolar Disorders*			
Dutch-born	1,008 (2.40)	1 (REF)	1 (REF)
Conflict affected	21 (1.81)	0.74 (0.48–1.14)	0.74 (0.48–1.14)
Non-conflict affected	73 (3.24)	1.28 (1.01–1.62)	1.20 (0.95–1.53)
*Psychotic Disorders*			
Dutch-born	1,419 (3.38)	1 (REF)	1 (REF)
Conflict affected	164 (14.12)	4.14 (3.52–4.87)	3.99 (2.39–4.69)
Non-conflict affected	157 (6.96)	1.99 (1.68–2.34)	2.03 (1.72–2.34)
*Alcohol Related Disorders*			
Dutch-born	1,185 (2.82)	1 (REF)	1 (REF)
Conflict affected	25 (2.15)	0.76 (0.51–1.13)	0.73 (0.49–1.09)
Non-conflict affected	37 (1.64)	0.58 (0.42–0.80)	0.59 (0.42–0.81)
*Substance Related Disorders*			
Dutch-born	4,419 (10.52)	1 (REF)	1 (REF)
Conflict affected	105 (7.69)	0.70 (0.56–0.86)	0.68 (0.55–0.84)
Non-conflict affected	344 (7.86)	0.59 (0.50–0.70)	0.63 (0.53–0.74)
*Somatoform Disorders*			
Dutch-born	2,874 (6.84)	1 (REF)	1 (REF)
Conflict affected	40 (4.30)	0.63 (0.48–0.83)	0.63 (0.47–0.83)
Non-conflict affected	40 (1.77)	0.26 (0.19–0.35)	0.23 (0.17–0.32)

^a^Model 1: Crude

^b^Model 2: Adjusted for Age (baseline) and sex

### Exploratory analyses

Table 3 shows the associations between migration background and specialized mental health care utilization, stratified by benefit receipt at baseline. Among individuals receiving benefits as the primary income, both risk of utilization of specialized mental health care was less for any disorder compared to Dutch-born individuals, with a larger reduction among conflict-affected migrants (aHR 0.45, 95% CI 0.42–0.49) than non-conflict-affected migrants (aHR 0.72, 95% CI 0.66–0.79). Among those not reliant on benefits, differences were smaller for any disorder (conflict-affected migrants: aHR 0.92, 95% CI 0.88–0.97; non-conflict-affected migrants: aHR 0.64, 95% CI 0.62–0.66). For psychotic disorders, both migrant groups had higher utilization than Dutch-born individuals, regardless of benefit receipt. The highest risk was observed among conflict-affected migrants not reliant on benefits (aHR 4.37, 95% CI 3.53–5.42). For anxiety disorders, there was no risk of increased utilization among migrants reliant on benefits compared to the Dutch-born population. Among those not reliant on benefits, conflict-affected migrants had slightly elevated risk for utilization (aHR 1.21, 95% CI 1.10–1.33), while non-conflict-affected migrants had a lower risk (aHR 0.68, 95% CI 0.63–0.74) compared to the Dutch-born group.

**Table 3:** Specialized mental health care utilization, by migration background and benefit receipt, among individuals aged 18–25 residing in the Netherlands in 2015. Hazard ratios (HRs) with 95% confidence intervals (CIs)

	Receiving benefits (*n* = 68,267)			Not receiving benefits (*n* = 1,150,784)		
	*N* (rate per 10,000 person-years)	Model 1^a^	Model 2^b^	*N* (rate per 10,000 person-years)	Model 1^a^	Model 2^b^
*Any diagnosis*						
Dutch-born	14,583 (711.23)	1 (REF)	1 (REF)	74,715 (186.90)	1 (REF)	1 (REF)
Conflict affected	567 (317.92)	0.45 (0.41–0.49)	0.45 (0.41–0.49)	1,693 (171.14)	0.92 (0.87–0.96)	0.92 (0.88–0.97)
Non-conflict affected	507 (526.57)	0.73 (0.67–0.80)	0.72 (0.66–0.79)	2,676 (123.92)	0.65 (0.63–0.68)	0.64 (0.62–0.66)
*Depressive Disorders*						
Dutch-born	1,207 (58.87)	1 (REF)	1 (REF)	13,695 (34.26)	1 (REF)	1 (REF)
Conflict affected	60 (33.64)	0.57 (0.44–0.74)	0.57 (0.44–0.74)	322 (32.74)	0.95 (0.85–1.06)	0.97 (0.86–1.08)
Non-conflict affected	64 (66.47)	1.12 (0.87–1.44)	1.05 (0.81–1.34)	544 (25.19)	0.73 (0.67–0.80)	0.70 (0.64–0.76)
*Anxiety Disorders*						
Dutch-born	2,389 (116.51)	1 (REF)	1 (REF)	15,253 (38.16)	1 (REF)	1 (REF)
Conflict affected	167 (93.64)	0.81 (0.69–0.94)	0.81 (0.69–0.95)	449 (45.65)	1.19 (1.09–1.31)	1.21 (1.10–1.33)
Non-conflict affected	94 (97.63)	0.83 (0.67–1.02)	0.77 (0.62–0.94)	603 (27.92)	0.72 (0.67–0.79)	0.68 (0.63–0.74)
*Psychotic Disorders*						
Dutch-born	587 (28.63)	1 (REF)	1 (REF)	832 (2.08)	1 (REF)	1 (REF)
Conflict affected	72 (40.37)	1.41 (1.11–1.80)	1.39 (1.09–1.78)	92 (9.35)	4.44 (3.58–5.51)	4.37 (3.53–5.42)
Non-conflict affected	56 (58.16)	2.02 (1.53–2.65)	2.15 (1.63–2.83)	101 (4.68)	2.15 (1.75–2.65)	2.30 (1.87–2.84)
*Substance Related Disorders*						
Dutch-born	996 (48.58)	1 (REF)	1 (REF)	3,423 (8.56)	1 (REF)	1 (REF)
Conflict affected	24 (13.46)	0.28 (0.19–0.42)	0.28 (0.18–0.42)	62 (6.30)	0.73 (0.57–0.94)	0.72 (0.56–0.92)
Non-conflict affected	29 (30.12)	0.61 (0.42–0.89)	0.66 (0.45–0.95)	116 (5.37)	0.61 (0.51–0.74)	0.66 (0.55–0.80)

^a^Model 1: Crude

^b^Model 2: Adjusted for Age (baseline) and sex

Table 4 depicts the associations between the duration of stay in the Netherlands and the utilization of specialized mental health care. Among conflict-affected migrants, those who had lived in the Netherlands for over 10 years had similar utilization rates that were comparable to Dutch-born individuals for any disorder (aHR 0.96, 95% CI 0.91–1.02). In contrast, non-conflict affected migrants with more than 10 years of stay showed a higher risk of using specialized mental health care compared to the Dutch-born group (aHR 1.22, 95% CI 1.16–1.28) for any disorder. The risk of using specialized mental health care for anxiety disorders was higher than that of the Dutch-born group for conflict-affected migrants with the shortest duration of stay (aHR 1.60, 95% CI 1.42–1.80). For psychotic disorders, the risk of utilization increased substantially with longer stays, particularly among conflict-affected migrants (aHR 4.43, 95% CI 3.59–5.46) and non-conflict-affected migrants (aHR 4.64, 95% CI 3.73–5.78) compared to the Dutch-born group.

**Table 4:** Specialized mental health care utilization, by migration background and duration of stay in the Netherlands, among individuals aged 18–25 residing in the Netherlands in 2015. Hazard ratios (HRs) with 95% confidence intervals (CIs)

	*N* (rate per 10,000 person-years)	Model 1^a^	Model 2^b^
*Any Diagnosis*			
Dutch-born	89,298 (212.49)	1 (REF)	1 (REF)
*Conflict Affected Migrants*			
Duration of stay: 3–5 years	750 (180.59)	0.84 (0.78–0.90)	0.85 (0.79–0.91)
Duration of stay: 6–10 years	288 (188.71)	0.88 (0.78–0.99)	0.88 (0.78–0.99)
Duration of stay: >10 years	1,222 (205.74)	0.96 (0.91–1.02)	0.96 (0.91–1.02)
*Non-Conflict Affected Migrants*			
Duration of stay: 3–5 years	1,251 (86.78)	0.39 (0.37–0.42)	0.38 (0.36–0.40)
Duration of stay: 6–10 years	478 (184.93)	0.86 (0.79–0.94)	0.86 (0.78–0.94)
Duration of stay: >10 years	1,454 (261.66)	1.23 (1.16–1.29)	1.22 (1.16–1.28)
**Depressive Disorders**			
Dutch-born	14,902 (35.46)	1 (REF)	1 (REF)
*Conflict Affected Migrants*			
Duration of stay: 3–5 years	117 (28.17)	0.80 (0.66–0.95)	0.81 (0.68–0.97)
Duration of stay: 6–10 years	45 (29.49)	0.83 (0.62–1.11)	0.83 (0.62–1.11)
Duration of stay: >10 years	22 (37.03)	1.04 (0.91–1.19)	1.05 (0.92–1.19)
*Non-Conflict Affected Migrants*			
Duration of stay: 3–5 years	298 (20.67)	0.58 (0.51–0.64)	0.54 (0.48–0.60)
Duration of stay: 6–10 years	79 (30.56)	0.86 (0.69–1.07)	0.85 (0.68–1.06)
Duration of stay: >10 years	231 (41.57)	1.17 (1.03–1.33)	1.16 (1.01–1.32)
**Anxiety Disorders**			
Dutch-born	17,642 (41.98)	1 (REF)	1 (REF)
*Conflict Affected Migrants*			
Duration of stay: 3–5 years	275 (66.21)	1.57 (1.39–1.77)	1.60 (1.42–1.80)
Duration of stay: 6–10 years	76 (49.80)	1.18 (0.94–1.47)	1.19 (0.95–1.48)
Duration of stay: >10 years	265 (44.62)	1.06 (0.94–1.20)	1.06 (0.94–1.20)
*Non-Conflict Affected Migrants*			
Duration of stay: 3–5 years	306 (21.22)	0.50 (0.44–0.55)	0.45 (0.40–0.50)
Duration of stay: 6–10 years	119 (46.04)	1.09 (0.91–1.31)	1.08 (0.90–1.28)
Duration of stay: >10 years	272 (48.95)	1.16 (1.03–1.31)	1.15 (1.02–1.29)
**Psychotic Disorders**			
Dutch-born	1419 (3.38)	1 (REF)	1 (REF)
*Conflict Affected Migrants*			
Duration of stay: 3–5 years	48 (11.56)	3.36 (2.52–4.48)	3.22 (2.41–4.29)
Duration of stay: 6–10 years	23 (15.07)	4.39 (2.91–6.63)	4.27 (2.83–6.45)
Duration of stay: >10 years	93 (15.66)	4.60 (3.73–5.68)	4.43 (3.59–5.46)
*Non-Conflict Affected Migrants*			
Duration of stay: 3–5 years	36 (2.50)	0.70 (0.50–0.97)	0.70 (0.50–0.98)
Duration of stay: 6–10 years	36 (13.93)	4.07 (2.93–5.67)	4.18 (3.00–5.82)
Duration of stay: >10 years	85 (15.30)	4.49 (3.61–5.59)	4.64 (3.73–5.78)

^a^Model 1: Crude

^b^Model 2: Adjusted for Age (baseline) and sex

Visual inspection of Schoenfeld residual plots for the exploratory analyses showed no evidence of violation of the proportional hazards assumption.

### Sensitivity analyses

When restricting the sample to migrants from low- and middle-income countries (LMICs), both conflict-affected and non-conflict-affected migrants continued to show lower overall utilization of specialized mental health care compared to Dutch-born individuals, although conflict-affected migrants had higher risks for anxiety and psychotic disorders (Supplementary Table 2). Among migrants only, conflict-affected individuals showed higher utilization than non–conflict-affected migrants in crude and age- and sex-adjusted models, but the difference was attenuated after additionally adjusting for country of birth (Model 3; Supplementary Table 3). Lastly, analysis limited to emergency admission diagnoses indicated slightly higher risks for both migrant groups versus Dutch-borns (Supplementary Table 4).

## Discussion

This study examined specialized mental health care utilization among young adults (18–25 years) in the Netherlands, focusing on differences by migration background. We compared conflict-affected migrants, non-conflict-affected migrants, and Dutch-born peers, and assessed variation by benefit receipt, and duration of stay. Our findings indicate that, overall, both migrant groups had lower rates of utilizing specialized mental health care than Dutch-born individuals. However, this pattern showed differences across diagnostic categories.

### Diagnosis-specific findings

All analyses in this study rely on service-recorded diagnoses as indicators of specialized mental health care utilization, and we acknowledge that diagnostic practices may vary. Despite lower overall utilization, both migrant groups showed higher use of specialized mental health care for psychotic disorders, consistent with prior research [12, 14, 40]. Conflict-affected migrants also more often utilized care for anxiety disorders. This finding may partly reflect diagnoses of PTSD, which are recorded under anxiety disorders in our data by specialized services and are known to be prevalent among refugees [6–8]. Because PTSD cannot be distinguished as a separate category in the registry, we were unable to examine PTSD-specific service use.

For bipolar disorders, non-conflict affected migrants showed a modest elevation in utilization compared with Dutch-born peers, but estimates were imprecise, encompassing both no difference and at most a modest increase in utilization. Given the number of diagnostic categories examined, this finding should be interpreted cautiously. While evidence for elevated psychotic disorder risk among migrants is consistent, findings for bipolar disorder (especially non-psychotic bipolar disorder) have been less extensively studied and remain mixed [41–43]. A previous meta-analysis found that while migrants had an elevated risk of bipolar disorder, it was lower than the risk for psychotic disorders [44]. Moreover, a population-based study from the Netherlands, which identified patients at their first contact with a physician for psychotic disorders and conducted diagnostic interviews, found that the risk of psychotic disorders was higher for adult migrants, while this was not the case for bipolar disorders [45]. On the other hand, a study from the United Kingdom found significantly higher incidence rates of bipolar disorder in certain migrant and ethnic minority groups, particularly in urban areas with greater socioeconomic deprivation and residential mobility [46]. These findings suggest that contextual factors, such as socioeconomic environment and mobility, could shape the risk of mood disorders across different migrant populations. Studies also suggest that age at migration is inversely associated with the risk of bipolar disorder, with younger age at migration linked to a higher risk [47]. Psychotic symptoms are a common feature of bipolar disorder, especially in bipolar I, where they often emerge during mood episodes. A systematic review of 339 studies reported that more than half of individuals with bipolar disorder experienced psychosis at some point, with such symptoms being notably more prevalent in bipolar I than in bipolar II [48]. Differences in classification systems and diagnostic criteria may partly explain the inconsistencies in findings across studies. Our study utilized DSM-IV classifications for bipolar disorder, including bipolar with psychosis. In contrast, other studies employed International Classification of Diseases (ICD) [49] criteria [12, 43, 47], non-psychotic bipolar disorder diagnoses [40], or broader categorizations of affective disorders [45]. Taken together, the present findings do not allow firm conclusions regarding bipolar disorder risk among migrants and highlight the need for further research using harmonized diagnostic definitions and designs that can better address heterogeneity and uncertainty.

### The role of socioeconomic and migration-related factors

Variation in specialized mental health care utilization was associated with several individual-level factors, one of which was benefit receipt. To better understand its role, we conducted exploratory analyses for individuals whose income was dependent on the benefits and who were not reliant on social benefits. Both migrant groups had higher risks of psychotic disorders than Dutch-born peers, regardless of benefit status. Notably, conflict-affected migrants not receiving benefits had over four times the risk, suggesting employment-related stressors or other vulnerabilities beyond benefit status influence care use. A previous study from the Netherlands found that migrants from groups reporting higher levels of perceived discrimination had the greatest risk of developing psychotic disorders [45]. Migrants engaged in the workforce may face greater exposure to discrimination and social exclusion in professional and social environments, potentially elevating this risk. Additionally, among conflict-affected migrants, those receiving benefits were less likely to utilize specialized care for anxiety disorders compared to those with other income sources. These findings highlight the need to consider broader social and economic factors driving mental health disparities among migrants.

Duration of stay in the Netherlands was another factor investigated in the study. Findings showed that, in line with previous research [25], longer residence was associated with increased utilization of specialized care across almost all of the diagnoses reported. This could indicate cumulative exposure to structural disadvantages in the host country or better access to specialized services over time. It is also possible that lower trust in institutions or stronger mental health–related stigma among some migrant groups, particularly in the initial phase after migration, contributes to lower use earlier after arrival. Both lack of trust and stigma may reduce over time. When restricting the sample to migrants from non-conflict-affected LMICs, their utilization risk became comparable to that of conflict-affected migrants. However, adjusting for country of birth likely accounted for variations in psychiatric risk. Youth in LMICs are particularly vulnerable to mental health stressors such as unemployment, food insecurity, and other adversities [50]. While conflict-affected migrants may have faced more severe or prolonged conflicts, our findings suggest that psychiatric risk may not be solely driven by conflict exposure but rather shaped by broader country-specific factors influencing both mental health and healthcare access.

### Utilization and barriers

As our study cannot separate mental health needs from care use, it’s unclear whether lower specialized care rates among migrants reflect lower prevalence, underdiagnosis, or access barriers. Prior research points to factors such as stigma, limited system knowledge, and language difficulties [10, 51], which may explain the lower utilization, as these barriers can delay help-seeking until a crisis occurs. Supporting this, our sensitivity analysis excluding non-emergency contacts showed higher utilization among migrants, suggesting greater reliance on acute care pathways. In the Netherlands, access to specialized care requires a GP referral, which may pose challenges for migrants lacking GP registration or familiarity with the system. Furthermore, cultural differences in symptom expression may affect recognition of psychological issues, as suggested in recent studies [4], which might impact the rate of referrals to specialized care.

### Strengths and limitations

This register-based study, covering over 1.2 million individuals, offers important insights into disparities in specialized mental health care utilization among young adults in the Netherlands. The use of large-scale national administrative data and the ability to analyze a broad range of diagnostic categories were the key strengths. Additionally, incorporating important socioeconomic and migration-related factors enhanced the interpretation of the findings. Nonetheless, several limitations should be considered. First, our primary outcome was limited to the first instance of a registered diagnosis and treatment combination (i.e., DBC). While this indicates that care was initiated and a diagnosis was recorded, we did not use additional data to determine whether treatment was subsequently provided or what type of treatment occurred. As such, our outcome does not allow us to distinguish between need, access, or the extent and nature of care received. Second, our data excluded mild-to-moderate cases likely managed in primary care, as we only accessed specialized mental health care records. Third, due to incomplete data on migration motives, we classified migrants as conflict-affected or non-conflict-affected based on their country of birth. This may have led to misclassification, as some individuals from non-conflict-affected countries may have sought asylum for other security-related reasons. Fourth, the completeness of the specialized mental health care registry declined sharply after 2018 due to changes in the Health Insurance Act, which affected the reporting of psychiatric care utilization [34], which led us to focus on the period between 2015 and 2018 and failed to capture more recent migration influxes. Moreover, no formal validity studies have been conducted on this registry. Additionally, although educational attainment is often used as a socioeconomic indicator in studies of mental health care use [25] we were unable to include it due to the large proportion of missing data, particularly among migrants who completed their education outside the Netherlands. Considering the young age of the cohort (16–25 years), many participants had not yet completed their education, which further limited the interpretability of the results in our context. For these reasons, we did not include education in our analyses, which may reduce the comparability of our results with studies that adjust for this variable. Lastly, we excluded individuals with prior specialized mental health care use before baseline. This approach allowed us to focus on first-time care users but may have introduced selection bias, particularly among migrants with longer durations of stay. Although our findings regarding duration of stay are consistent with previous research, it is possible that we captured a comparatively ‘healthier’ subgroup among long-term residents, as those with earlier or more severe onset were excluded. This may have attenuated the observed association between duration of stay and specialized care utilization.

## Conclusion

Young adults with a migration background (both conflict-affected and non-conflict-affected) used specialized mental health care less often than their Dutch-born peers, though rates were higher for some diagnoses, such as psychotic and anxiety disorders. Utilization patterns also varied by socioeconomic and structural characteristics, namely benefit status and duration of stay in the Netherlands. These variations may reflect a combination of factors, including structural obstacles, limited system knowledge, language barriers, and financial constraints, though our data do not allow us to determine the exact mechanisms. Enhancing health system navigation, provider cultural competency, and migrant integration could improve access. Future research should explore how mental health needs translate into care and include primary care data to detect early disparities and to improve access before conditions worsen.

## Supplementary Information

Below is the link to the electronic supplementary material.

Supplementary Material 1

## Data Availability

Results are based on calculations by the authors using non-public microdata fromStatistics Netherlands. Under certain conditions, these microdata are accessible for statistical and scientific research. For further information: [https://www.cbs.nl/en-gb/our-services/customised-services-microdata/microdata-conducting-your-own-research](https:/www.cbs.nl/en-gb/our-services/customised-services-microdata/microdata-conducting-your-own-research).

## Footnotes

1 The list of conflict-affected countries was based on the Uppsala Universitet. (n.d.). Uppsala Conflict Data Program. Retrieved December 11, 2024, https://ucdp.uu.se/encyclopedia.
