# Supplementary material for: Migration background and specialized mental health care utilization in young adults: a register-based study in the Netherlands
Source: Soc Psychiatry Psychiatr Epidemiol. 2026 Feb 11;61(10):2125–36. doi: 10.1007/s00127-026-03052-0 (PMC13585872; doi:10.1007/s00127-026-03052-0)
Supplement: Supplementary file 1 [file 127_2026_3052_MOESM1_ESM.docx]

**Supplementary Materials**

Supplementary Table 1. FDR-corrected results for the primary Cox regression models across diagnostic categories

| Diagnosis category | Migration Group | Raw p-value | FDR adjusted p-value |
| --- | --- | --- | --- |
| Any Disorders | Conflict Affected | 0.000 | 0.000 |
|  | Non-Conflict Affected | 0.000 | 0.000 |
| Depressive Disorders | Conflict Affected | 0.187 | 0.187 |
|  | Non-Conflict Affected | 0.000 | 0.000 |
| Anxiety Disorders | Conflict Affected | 0.000 | 0.000 |
|  | Non-Conflict Affected | 0.000 | 0.000 |
| Bipolar Disorders | Conflict Affected | 0.171 | 0.182 |
|  | Non-Conflict Affected | 0.131 | 0.150 |
| Psychotic Disorders | Conflict Affected | 0.000 | 0.000 |
|  | Non-Conflict Affected | 0.000 | 0.000 |
| Alcohol Related Disorders | Conflict Affected | 0.120 | 0.147 |
|  | Non-Conflict Affected | 0.001 | 0.002 |
| Substance Related Disorders | Conflict Affected | 0.000 | 0.001 |
|  | Non-Conflict Affected | 0.000 | 0.000 |
| Somatoform Disorders | Conflict Affected | 0.001 | 0.002 |
|  | Non-Conflict Affected | 0.000 | 0.000 |

Supplementary Table 2. Specialized mental health care utilization, by migration background (only migrants from LMICs). Hazard ratios (HRs) with 95% confidence intervals (CIs).

|  | Model 1^a^ | Model 2^b^ |
| --- | --- | --- |
| Any diagnosis |  |  |
| Dutch-born | 1 (REF) | 1 (REF) |
| Conflict affected | **0.91 (0.87-0.95)** | **0.91 (0.88-0.95)** |
| Non-conflict affected | **0.71 (0.68-0.75)** | **0.70 (0.66-0.73)** |
| Depressive Disorders |  |  |
| Dutch-born | 1 (REF) | 1 (REF) |
| Conflict affected | 0.92 (0.84-1.02) | 0.97 (0.84-1.03) |
| Non-conflict affected | **0.79 (0.70-0.88)** | **0.76 (0.68-0.84)** |
| Anxiety Disorders |  |  |
| Dutch-born | 1 (REF) | 1 (REF) |
| Conflict affected | **1.26 (1.16-1.36)** | **1.27 (1.17-1.37)** |
| Non-conflict affected | **0.76 (0.68-0.84)** | **0.72 (0.65-0.80)** |
| Bipolar Disorders |  |  |
| Dutch-born | 1 (REF) | 1 (REF) |
| Conflict affected | 0.74 (0.48-1.14) | 0.74 (0.48-1.14) |
| Non-conflict affected | **1.43 (1.04-1.96)** | **1.36 (0.99-1.87)** |
| Psychotic Disorders |  |  |
| Dutch-born | 1 (REF) | 1 (REF) |
| Conflict affected | **4.14 (3.52-4.87)** | **3.98 (3.39-4.68)** |
| Non-conflict affected | **2.67 (2.19-3.25)** | **2.72 (2.23-3.32)** |
| Alcohol Related Disorders |  |  |
| Dutch-born | 1 (REF) | 1 (REF) |
| Conflict affected | 0.76 (0.51-1.13) | 0.73 (0.49-1.08) |
| Non-conflict affected | 0.72 (0.42-0.80) | 0.73 (0.48-1.10) |
| Substance Related Disorders |  |  |
| Dutch-born | 1 (REF) | 1 (REF) |
| Conflict affected | **0.70 (0.56-0.86)** | **0.68 (0.55-0.84)** |
| Non-conflict affected | **0.62 (0.49-0.78)** | **0.65 (0.52-0.82)** |
| Somatoform Disorders |  |  |
| Dutch-born | 1 (REF) | 1 (REF) |
| Conflict affected | **0.63 (0.48-0.83)** | **0.63 (0.47-0.83)** |
| Non-conflict affected | **0.26 (0.17-0.40)** | **0.24 (0.15-0.37)** |

^a^Model 1: Crude

^b^Model 2: Adjusted for baseline age and sex

*Statistically significant hazard ratios (HRs) are shown in bold text (p < 0.05)*

Supplementary Table 3. Specialized mental health care utilization for any psychiatric diagnosis, by migration background (only migrants, excluding the Dutch-borns)

| Migration background | Model 1^a^ | Model 2^b^ | Model 3^c^ |
| --- | --- | --- | --- |
| Non-Conflict Affected | 1 (REF) | 1 (REF) | 1 (REF) |
| Conflict-Affected | 1.39 (1.32-1.47) | 1.41 (1.34-1.49) | 1.15 (1.04-1.27) |

^a^Model 1: Crude

^b^Model 2: Adjusted for baseline age and sex.

^c^Model 3: Adjusted for baseline age, sex, and country of birth.

Supplementary Table 4. Specialized mental health care utilization, by migration background (only emergency admissions). Hazard ratios (HRs) with 95% confidence intervals (CIs).

|  | Model 1^a^ | Model 2^b^ |
| --- | --- | --- |
| Any diagnosis |  |  |
| Dutch-born | 1 (REF) | 1 (REF) |
| Conflict affected | 1.09 (0.99 -1.19) | 1.09 (1.00-1.19) |
| Non-conflict affected | 1.08 (1.01 -1.16) | 1.08 (1.01-1.15) |

^a^Model 1: Crude

^b^Model 2: Adjusted for baseline age and sex.

Supplementary Table 5. Conflict-affected migrants country list

| Country | |
| --- | --- |
| Afghanistan | Congo (Republic of the Congo) |
| Algeria | Laos |
| Angola | Lebanon |
| Armenia | Liberia |
| Azerbaijan | Libya |
| Bhutan | Mali |
| Bosnia and Herzegovina | Mauritania |
| Burundi | Mongolia |
| Cambodia | Myanmar |
| Central African Republic | Nepal |
| Chad | Niger |
| Democratic Republic of the Congo | Nigeria |
| Djibouti | North Korea |
| Eritrea | Pakistan |
| Ethiopia | Russia |
| Federal Republic of Yugoslavia | Rwanda |
| Gabon | Sierra Leone |
| Georgia | Somalia |
| Ghana | Soviet Union (Former) |
| Guinea | Sudan |
| Guinee-Bissau | Syria |
| Iran | Tajikistan |
| Iraq | Togo |
| Ivory Coast (Côte d'Ivoire) | Uganda |
| Jordan | Yemen |
| Kazakhstan | Yugoslavia (Former) |
| Kenya | Zaire (now Democratic Republic of the Congo) |
| Kyrgyzstan | Zimbabwe |

Supplementary Table 6. DSM-IV diagnoses studied

| DIS categories | Main Diagnosis |
| --- | --- |
| asl_6 (as1_6.01) | Depressive Disorders |
| as1_7 (as1_7.01-as1_7.12) | Anxiety Disorders |
| as1_6 (as1_6.02) | Bipolar Disorder |
| as1_5 (as1_5.01-as1_5.07) | Psychotic Disorders |
| as1_4 (as1_4.01) | Alcohol Related Disorders |
| as1_4 (as1_4.02-as1_4.13) | Substance Related Disorders |
| as1_15 (as1_15.01) | Adjustment Disorders* |
| as1_8 (as1_8.01-as1_8.08) | Somatoform Disorders |

*Results of adjustment disorder analyses are not reported due to low occurrences, in line with CBS Microdata usage rules

Supplementary Table 7. Primary Source of Income categories

| Categories in the registers | Primary Source of Income |
| --- | --- |
| Employee (salary-based) | Other than benefits (0) |
| Major shareholder | Other than benefits (0) |
| Self-employed | Other than benefits (0) |
| Recipient of unemployment benefits | Benefits (1) |
| Recipient of social assistance benefits | Benefits (1) |
| Recipient of other social security benefits | Benefits (1) |
| Recipient of sickness and disability benefits | Benefits (1) |
| Recipient of pension benefits | Benefits (1) |
| Student with income | Other than benefits (0) |
| Student without income | Other than benefits (0) |
| Other without income | Other than benefits (0) |
